# Selenium Enrichment of the Edible Medicinal Mushroom *Antrodia camphorata* by Submerged Fermentation

**DOI:** 10.3390/molecules28073036

**Published:** 2023-03-29

**Authors:** Jin Li, Sai Wen, Baoyuan Zhang, Fenghuan Wang

**Affiliations:** School of Light Industry, Beijing Technology and Business University (BTBU), Beijing 100048, China

**Keywords:** *Antrodia camphorata*, selenium enrichment, polysaccharide, submerged fermentation

## Abstract

Selenium (Se) is an essential nutrient element in human physiological metabolism and immune function. Supplementation of bioavailable Se will confer benefit on human life, especially when intake of this nutrient is inadequate. The edible and medicinal mushroom *Antrodia camphorata* is a unique fungus endemic to Taiwan, which has shown high therapeutic and nutritive value. This study is the first to demonstrate that *A. camphorata* can assimilate and transform sodium selenite into organic selenium. With an initial concentration of Se (IV) at 10 mg/L in 100 mL of the medium at 25 °C, the total selenium content in Se-enriched *A. camphorata* mycelia was 1281.3 ± 79.2 µg/g, in which the organic selenium content accounted for 88.1%. Further analysis demonstrated that selenium-enriched polysaccharide was the main form of Se present in *A. camphorata* (61.5% of the organic selenium). Four water-soluble Se-polysaccharide fractions were separated from *A. camphorata*, and ACP II was the major fraction of Se-polysaccharide. The scavenging efficiency of Se-polysaccharides on DPPH and ABTS radicals was determined, proving that selenium enrichment dramatically improved the in vitro antioxidant capacity of *A. camphorata* polysaccharide. Therefore, the selenium accumulation and transformation ability of *A. camphorata* provides an opportunity for developing this beneficent fungus into a novel selenium-enriched dietary or medicinal supplement.

## 1. Introduction

Selenium is an essential trace element [1], which is the basic component of the active site of some vital enzymes (such as glutathione peroxidase and thioredoxin reductase) and has important physiological effects [2]. Selenium in the form of selenocysteine is present in the active center of glutathione peroxidase, which task is to reduce hydrogen peroxide and organic peroxides [3]. Selenoproteins 5′DI, 5′DII, and 5′DIII are found primarily in the liver, kidneys, thyroid, and brown fat, and they play a role in thyroid hormone metabolism [4]. In the immune response, selenium has been shown to stimulate the antibody formation and activity of helper T cells, along with cytotoxic T and NK cells. It is also implicated in the stimulation of phagocytic cell migration and in phagocytosis [4]. Therefore, selenium deficiency can cause a range of diseases, such as heart disease, cancer, liver disease, immune system disorders, anemia, liver cirrhosis, diabetes, and Kashin–Beck disease (KBD) [5,6,7,8]. The human body intakes selenium through dietary supplement, but the distribution of selenium in the world is extremely uneven. About 50 countries and regions have selenium deficiency problems due to low selenium bioavailability in the soil, and more than 70% of people in China are facing different degrees of selenium deficiency [9]. Inorganic selenium, such as sodium selenite (Na_2_SeO_3_) and sodium selenate, (Na_2_SeO_4_), though easily manufactured and applied as nutritional supplements, cannot be directly stored in general body proteins. By comparison, the absorptivity and bioactivity of organic Se are higher than those of inorganic Se; meanwhile, the toxicity of organic Se is much lower at high concentrations of intake. In selenium-rich foods, organic Se is the predominant form, and it is naturally transformed by plants and soil microorganisms. Till now, some edible fungi have been discovered to possess the ability for selenium enrichment [10], such as *Hericium erinaceus*, *Pleurotus ostreatus*, and *Agaricus bisporus* [11,12]. There are few studies, however, on the medicinal fungus *A. camphorata* on its accumulation and transformation of selenium.

*Antrodia camphorata* (*Syn. Antrodia cinnamomea*) is a fungus that is endemic to Taiwan, where it grows only on the inner cavity of the endemic tree Cinnamomum kanehirae [13]. In Taiwan, *A. camphorata* is a folk medicinal mushroom commonly used as a traditional Chinese medicine by the aborigines and has shown liver protection, anti-alcohol, anti-cancer, and anti-inflammatory effects [14,15,16]. Over 78 compounds have been identified and structurally elucidated in *A. camphorate* [16]. The most abundant active components of *A. camphorata* include polysaccharides [17], terpenoids (such as antcins C and K) [18], benzenoids, lignans, benzoquinone derivatives, and succinic and maleic derivatives [19]. Therefore, *A. camphorata* is a promising natural medicine. The polysaccharides produced by submerged fermentation of *A. camphorata* have been reported to possess potent biological activities, including anti-hepatitis B virus, anti-inflammatory, and anticancer effects [20]. For instance, a water-soluble *A. camphorata* polysaccharide, β-d-glucan, could alleviate oxidation and inflammation via the suppression of NADPH oxidase activation and the inhibition of the ERK, p38, and Akt signaling pathways [21]. In the past three decades, more than 160 terpenoids have been identified from *A. camphorata*. The potential of triterpenoids for the treatment of cancer, diabetes, and hyperlipidemia has been confirmed [22]. It is encouraging that *A. camphorata* shows good safety in both animal and human studies.

Submerged fermentation of *A. camphorata* mycelia provides an attractive alternative method to produce bioactive fungal metabolites with great economic value. The total market value of *A. camphorata* products is estimated to be over USD 100 million per year [23]. To satisfy the large consumption demand of *A. camphorata*, four artificial cultivation techniques can be adopted, i.e., submerged fermentation, solid support culture, cutting wood culture, and dish culture [24]. Submerged liquid cultivation has the benefits of reinforced oxygen mass transfer and culture homogeneity by agitation. Using this technique, physical (temperature, aeration, agitation, etc.), chemical (pH, medium composition, etc.), and biological (inoculum, morphology, and rheology) factors could be controlled [25]. Thus, submerged cultivation is a reproducible technique for mycelium production, while controlled yields of metabolites are achieved, making it easier for scale-up in industrial production [26]. Nowadays, production of mushroom bioactive compounds by submerged cultivation has attracted great interest, leading to the exploitation of mushroom mycelia as vegan protein sources, nutraceuticals, food supplements, and food flavor agents by the food industry [27]. Submerged fermentation has also been applied for manufacturing bioactive compounds of *A. camphorata* with high identity and purity [20]. The present study provided the first experimental evidence for *A. camphorata* as a good carrier for enriching organic selenium through submerged fermentation. Considering the potential anti-inflammatory and antioxidant properties of selenium [28], the current study is expected to increase the market value of *A. camphorata*.

This study aimed to address two main questions. The first aim was to demonstrate if *A. camphorata* could be used as a good carrier of selenium biofortification. The second aim was to determine the level of Se accumulation and Se-enriched components in *A. camphorata* cells after submerged fermentation. This work contributes to our knowledge of the therapeutic potential of the medicinal mushroom *A. camphorata* and provides new insights into its nutritional value as a novel Se-enriched food.

## 2. Results

### 2.1. Identification of Strain

**Figure 1:**
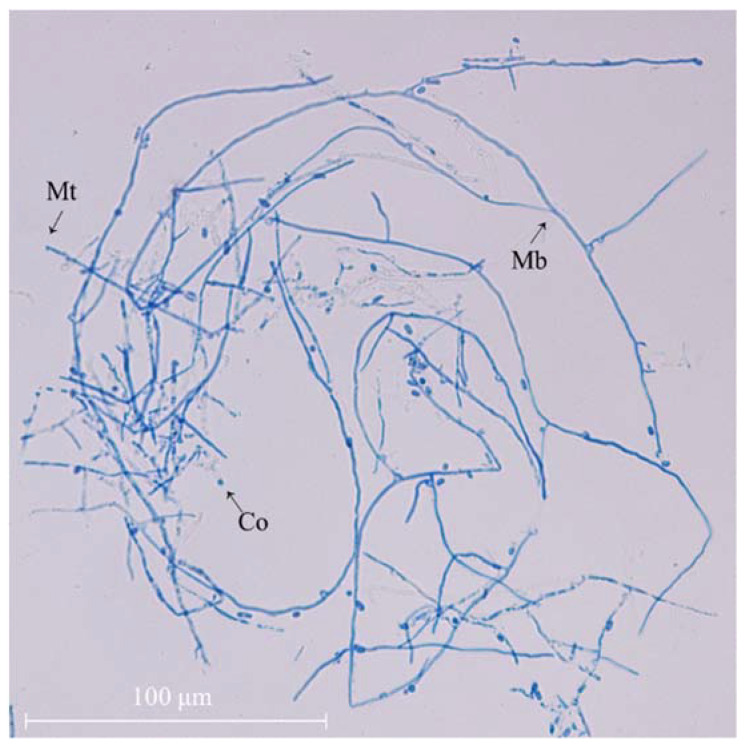
Mycelial morphology of *A. camphorata mycelia* (×40). Co, conidia; Mb, mycelium branch; Mt, mycelium tip.

The morphological features of mycelial *A. camphorata* was examined using an upright microscope (Figure 1). It can be seen that the uniformly dyed hyphae of *A. camphorata* are smooth and elongated continuously, with only a few conidia visible, after 15 days of growth.

**Figure 2:**
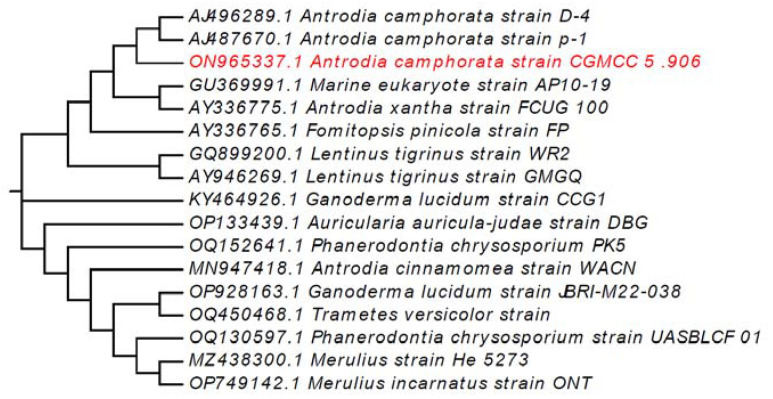
ITS gene sequence-generated phylogenetic tree.

As shown in Figure 2, the *A. camphorata* strain (GenBank: ON965337.1) was characterized by using ITS gene sequencing. Then, the alignment of the genetic sequences was obtained, and phylogenetic analysis was performed using the MEGA 11 with the neighbor-joining (NJ) method. According to the morphological and ITS molecular identification, our *A. camphorata* strain, CGMCC 5.906, is closely related to *Antrodia camphoratus* D-4 and p-1.

### 2.2. Selection of Selenium-Enriched Fermentation Conditions

#### 2.2.1. Effect of Temperature

**Figure 3:**
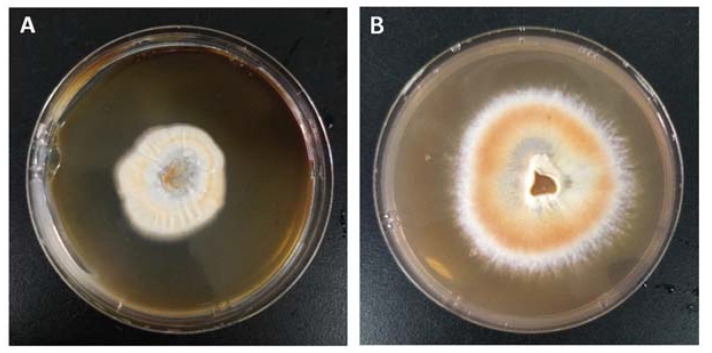
*A. camphorata* mycelia were cultured in solid media at different temperatures: (**A**) cultured at 30 °C and (**B**) cultured at 25 °C.

The effect of two temperatures mostly used for the cultivation of mushroom mycelia (i.e., 25 °C and 30 °C) on the growth state of *A. camphorata* were evaluated. *A. camphorata* mycelia were inoculated on a PDA solid medium and cultured separately at 25 °C and 30 °C for 15 days to observe the growth trend. As shown in Figure 3, the mycelia of *A. camphorata* on the culture dish have grown into a subrotund colony with a specific growth rate of about 0.35 cm/d at 25 °C, while the growth of the colony is much slower at 30 °C with a diameter around 0.14 cm/d. The appearance of the colony is also much darker at 25 °C, indicating that growing at a relatively lower temperature (25 °C) is conducive to the growth of mycelia and pigmentation. The reddish color of the mycelia is consistent with that of basidiocarps of wild *A. camphorata*, which generally indicates abundant metabolites of benzenoids and a higher biological activity [29]. Additional comparison was conducted in shake flasks by culturing *A. camphorata* mycelia in a liquid medium at these two temperatures for 10 days. The *A. camphorata* mycelia grew in this environment formed white spherical pellets. It showed that the final biomass accumulated to 1.55 ± 0.06 g/L and 1.45 ± 0.07 g/L at 25 °C and 30 °C, respectively.

To further examine the effect of temperature on the selenium-enrichment ability of *A. camphorata*, the biomass and total selenium content in the *A. camphorata* mycelia at these two temperatures were examined separately. As shown in Table 1, although the biomass (dry weight) obtained at 25 °C is only slightly higher, the concentration of total selenium in the dry mycelia at 25 °C is significantly higher, i.e., 1.32 times higher than that obtained at 30 °C. Our results suggest that *A. camphorata* is capable of accumulating selenium from the environment. The temperature control during the plate culture is critical since temperature can greatly influence the growth state of *A. camphorata* mycelia; in submerged culture, the influence of temperature on mycelial growth is minor, but it is significant on Se enrichment of *A. camphorata* (Table 1).

#### 2.2.2. Effect of Sodium Selenite Concentration

Selenite (Se (IV)) is often toxic due to its water solubility and bioavailability. To evaluate the effect of different concentrations of Se (IV) (i.e., 5, 10, 20, 40, and 80 mg Se (IV) /L) on the performance of Se enrichment of *A. camphorata*, the biomass and total Se content in the mycelia were determined.

**Figure 4:**
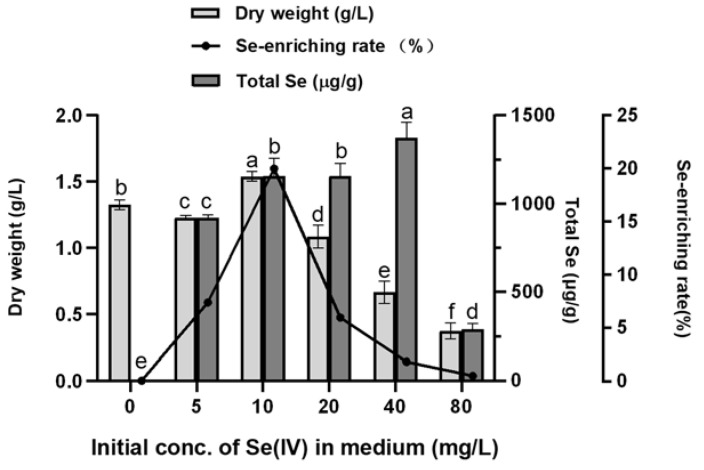
Biomass and selenium-enriching capacity of *A. camphorata* at different concentrations of initial Se (IV). Values with letters a, b, c, d, e, and f are significantly different across columns (*p* < 0.05).

According to Figure 4, when the concentration of inorganic selenium in the medium is below 10 mg/L, the biomass maintains a stable level and reaches the apex at a selenite concentration of 10 mg/L; it then decreases gradually with increasing concentrations of selenite. These results show that when Se concentration ≤10 mg/L, selenite slightly promotes the growth of *A. camphorata* mycelia, while when Se ≥20 mg/L, the growth of mycelia is inhibited in a dose-dependent manner. For the total content of selenium in *A. camphorate*, it increases gradually until the concentration of Se (IV) is over 40 mg/L, indicating that *A. camphorata* mycelia would be still able to enrich selenium when their growth is suppressed. To quantify the selenium-enriching capacity of *A. camphorata* mycelia, selenium-enriching rate was also calculated (Figure 4). The selenium-enriching rate shows the same tendency as the biomass, and the maximum rate is 20% at 10 mg/L. Then, the selenium-enriching rate decreases gradually and approaches zero when *A. camphorata* mycelia stop growing. These results demonstrate that the tolerance level of *A. camphorata* mycelia to selenite during submerged fermentation is around 10 mg/L, at which point both the selenium-enrichment of *A. camphorate* and the biomass reach the highest level.

#### 2.2.3. Effect of Liquid Filling Volume and Agitation

Fungal mycelia growing in a liquid medium create numerous mycelial spherical pellets, and the cells absorb oxygen and nutrients via diffusion [30]. The oxygen dissolved in the growth medium could affect hyphal elongation, cell wall properties, the roughness and size of pellets, and, therefore, the viscosity [31]. Therefore, the flask culture parameters of the liquid filling volume and rotation rate were examined for the effect on the selenium-enriching capacity and biomass of *A. camphorata* mycelia.

**Figure 5:**
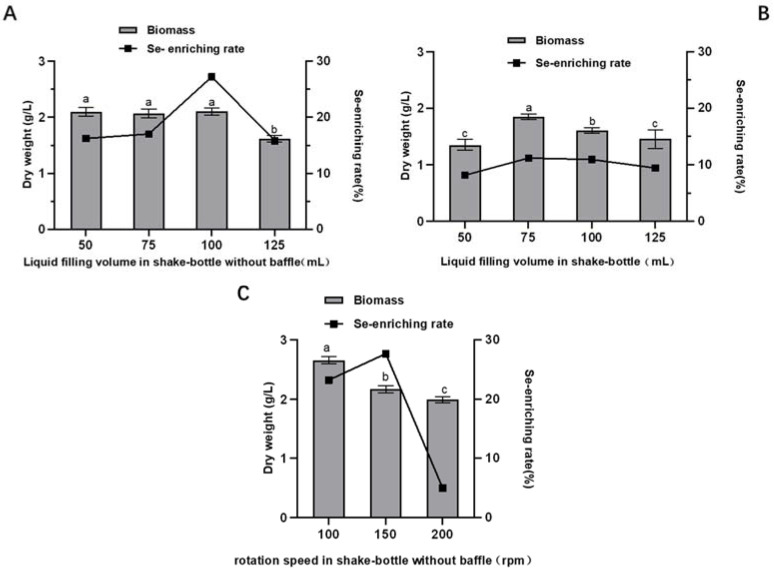
Effects of the liquid filling volume and rotational speed on the selenium-enriching capacity and the biomass in shake flask with or without baffle: (**A**) effect of liquid filling volume in shake flask without baffle; (**B**) effect of liquid filling volume in shake flask with baffle; and (**C**) effect of rotational speed in shake flask without baffle. Values with letters a, b and c are significantly different across columns (*p* < 0.05).

As depicted in Figure 5A, in the 250 mL shake flask without baffle, there is no obvious correlation between the biomass of *A. camphorata* and the liquid filling volume at the range of 50–100 mL, while a decrease of 24% in biomass is observed at a higher filling volume of 125 mL. In regard to the selenium-enriching capacity, when the filling volume is 100 mL, the selenium-enriching capacity of *A. camphorata* is highest, up to 27.23%. By contrast, in the shake flask with baffle, the selenium-enriching rate and the biomass are both much lower than those in the shake flask without the baffle at the same filling volume (Figure 5B). In this section, the liquid filling volume, baffle, and rotational speed are all factors that affect the oxygen transfer coefficient (Kla); however, the mycelium biomass does not have a good linear relation with the oxygen concentration. A possible explanation for this phenomenon is that the baffle in the flask, although it can increase turbulence and, thus, dissolved oxygen concentration, may cause the fragile *A. camphorata* mycelia to bear excess shear stress.

The influence of agitation rate on the biomass and the conversion rate of selenium were also determined. As shown in Figure 5C, the biomass reduces when the rotational speed increases, while the conversion rate of selenium first increases, reaching the highest point at 150 rpm, and then decreases. These results demonstrate that vigorous agitation can harm the growth of mycelia, and the Se-enriching efficacy of *A. camphorata* prefers a moderate agitation intensity, achieving a balance between oxygen transfer and shear stress. Overall, the biomass and the conversion rate of selenium in the shake flask without baffle are slightly higher than those obtained in the shake flask with baffle. Therefore, the better condition is to culture *A. camphorata* in 100 mL of the medium at 150 rpm in a shake flask without baffle.

### 2.3. Contents of Inorganic and Organic Selenium in A. camphorata

Selenium compounds can be divided into inorganic selenium (SeO_3_^2−^, SeO_4_^2−^, etc.) and organic selenium forms (selenoproteins, selenium polysaccharides, etc.). In the human body, selenium is incorporated at the active site of many selenoproteins, such as glutathione peroxidase, selenoprotein-S, and selenoprotein-P, where its crucial role as an antioxidant and in the regulation of inflammation has been widely investigated [32]. However, the gap between toxic and essential levels of Se in humans is narrow, and this is especially true for inorganic selenium [33]. Therefore, organic selenium compounds, which has a higher absorption rate and higher safety for ingestion, are more reliable and feasible as human nutritional supplement [34,35]. To clarify the transformation ability of selenium in *A. camphorata*, the contents of inorganic selenium and organic selenium in *A. camphorata* were determined, respectively, under the optimal selenium-enriching fermentation conditions (Table 2). The content of organic Se in *A. camphorata* is 1128.514 µg/g, which accounts for about 88.08% of the total selenium, indicating that *A. camphorata* could efficiently accumulate and transform inorganic Se into organic Se.

To demonstrate if fermentation is necessary for *A. camphorata* polysaccharide to assimilate and enrich selenium, incubation experiments of sodium selenite with purified selenium-free *A. camphorata* polysaccharide and without polysaccharide as a control were both tested. In the *A. camphorata* polysaccharide group, the final content of inorganic selenium is 8.76 mg/L, while in the control group, the content of inorganic selenium is 9.09 mg/L. Accordingly, there is no statistical difference between the two groups, indicating that fermentation is necessary for *A. camphorata* polysaccharide to accumulate and transform selenium. It also proves that only a minimal, if any at all, portion of inorganic selenium binds to polysaccharide so tightly that it cannot be extracted by the hydrochloric acid method.

In animals and plants, the organic selenium-rich components mainly include selenoprotein, selenium polysaccharide, and selenium tRNA. Given the instability of RNA, we only analyzed the content of selenium polysaccharide and selenoprotein in *A. camphorata*. As shown in Table 3, Se-polysaccharide and selenoprotein are the main forms of selenium presented in *A. camphorata*, which account for about 61.5% and 12.4% of the total selenium and about 54.2% and 10.9% of organic selenium, respectively. Selenium polysaccharide is the main form of organic selenium existing in selenium-enriched *A. camphorata*.

**Figure 6:**
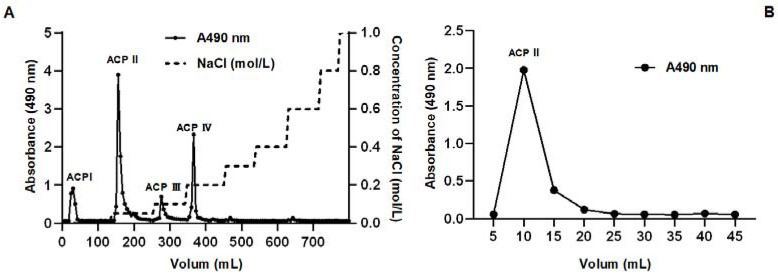
Fractionation of *A. camphorata* polysaccharides by ion-exchange chromatography and size-exclusion chromatography. Crude polysaccharides on HiTrap Q FF (**A**), and fraction II on Seuperdex 200 Increase 10/300 GL column (**B**).

Dried selenium-enriched polysaccharide dissolved in distilled water (10 mg/mL) was subjected to an ion-exchange chromatography with a 0–1 mol/L aqueous solution of NaCl as the elution buffer under the flow rate of 3 mL/min. The elute was collected and monitored at 490 nm for the measurement of the sugar content using the phenol–sulfuric acid method. As shown in Figure 6A, four polysaccharide fractions were obtained (named ACP I, ACP II, ACP III, and ACP IV). The yield and content of selenium of the ACP I, ACP II, ACP III, and ACP IV fractions were analyzed and summarized in Table 4. Although the content of selenium in the ACP IV fraction is the highest, the ACP II fraction is the major fraction with a slightly lower content of selenium. The fraction ACP II was then loaded onto a Superdex 200 column and eluted with 0.15 mol/L NaCl for further separation based on molecular weight. The elution diagram shows that the ACP II fraction is a homogeneous polysaccharide with a single, symmetrical narrow peak (Figure 6B).

Collectively, these results show that *A. camphorata* can intake, accumulate, and transform sodium selenite into Se-polysaccharide and selenoprotein, which is much safer for human as a nutritional supplement. Therefore, *A. camphorata* could be used as a good carrier of selenium biofortification, especially considering that the Se-polysaccharide from *A. camphorata* could combine nutritive and medicinal values of both selenium and polysaccharide.

### 2.4. In Vitro Antioxidant Activity of Polysaccharides

In this study, DPPH and ABTS, two widely used free radicals, were used as model substrates to explore the in vitro antioxidant capacity of Se-enriched *A. camphorata* polysaccharide (Se-ACP), with selenium-free *A. camphorata* polysaccharide (ACP) as a comparison, and ascorbic acid (Vc) as the positive control.

**Figure 7:**
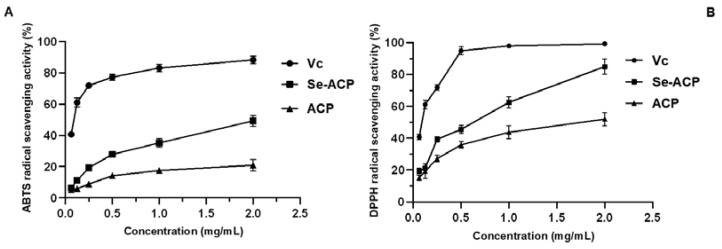
Antioxidant activity tests of ACP: (**A**) ABTS radical scavenging activity, and (**B**) DPPH radical scavenging activity.

The ABTS radical scavenging assay was mostly based on the electron transfer capacity of an antioxidant to reduce ABTS radicals, while the DPPH radical scavenging ability was based on the hydrogen-donating ability. As shown in Figure 7A, both the scavenging abilities of ACP and Se-ACP for ABTS radicals increase in a dose-dependent manner. At a concentration of 2.0 mg/mL, the scavenging ability of Se-ACP for ABTS radicals (up to 42.6%) is significantly higher than that of selenium-free *A. camphorata* polysaccharide (19.9%). Similarly, the DPPH radical scavenging capacities of Se-ACP and ACP both enhance with an increase in polysaccharide concentration in the test range (Figure 7B). Moreover, the DPPH radical scavenging ability of Se-ACP could reach up to 84.2% at the concentration of 2.0 mg/mL, which is 1.6 times higher than the activity of ACP (51.9%).

To sum up, by measuring the DPPH radical and ABTS radical scavenging abilities, it is found that Se enrichment contributes to the great enhancement in in vitro antioxidant capacity of *A. camphorata* polysaccharide.

## 3. Discussion

Thus far, in some mushroom species, depending upon their growth situation, the selenium form and concentration have shown different abilities to absorb and accumulate selenium [36,37]. In *Lentinula edodes* mycelia supplemented with selenium, considerable accumulation of this element (from 192.6 to 532.3 mg/100 g) was detected [38]. Total Se content in *Inonotus hispidus* grown with 0.29 mmol/L selenite could reach 532.3 µg/g [37]. With a Se concentration in the medium between 15.0 and 20.0 mg/L, the biomass of *Pleurotus ostreatus*, a common edible fungus, reached approximately 9.0 g/L, and the amount of selenium absorbed was about 800 µg/g [36]. Compared to other reported mushroom fungi, *A. camphorata* showed the highest selenium-enriching capacity (1281.3 ± 79.2 µg/g) with an initial concentration of Se (Ⅳ) at 10 mg/L.

It is worth noting that a high sodium selenite concentration in the medium is toxic and has a strong inhibitory effect on the growth of fungi. In a liquid culture of *Ganoderma lucidum*, another fungus in the polyporales order, a high concentration of Na_2_SeO_3_ elicited decreased biomass and degraded mycelial cells, leading to a red color and an unpleasant odor. The production of volatile selenium compounds is a self-detoxification mechanism that occurs under high selenite conditions, by which the concentration and toxicity of inorganic selenium compounds can be decreased for a better chance of survival [39]. As shown in this work, the biomass of *A. camphorata* mycelia, as well as its selenium-enriching rate, increased with an increasing concentration of selenite in the medium but decreased rapidly when the Se content exceeded 10 mg/L. However, it was observed that *A. camphorata* mycelia were still able to enrich selenium when their growth was suppressed at a Se concentration as high as 40 mg/L. Therefore, especially for fungi, the content of selenite in the medium is a crucial factor that has to be controlled to meet a balance between biomass accumulation and metabolic burden caused by selenium.

In most organisms, selenium enters the cells and forms selenmethionine (Se-Met) and selenocysteine (Sec). The discovered selenoproteins are multifunctional proteins and are commonly known as selenase (such as glutathione peroxidase (GPXs), thioredoxin reductase (TrxR), deiodinase (ID) and protease P), which can protect cells from oxidative damage caused by free radicals and exert great influence on the physiological functions of the human body [40]. In fact, owing to a 1996 clinical trial [41], nutritional selenium supplementation has become popular in recent years, and some of the most widely used selenium supplements are selenium-enriched yeasts. Se-enriched yeasts usually contain a high level of Se-methionine, which acts as a precursor for selenoprotein synthesis [42].

Unlike yeasts, *A. camphorata* was proved in this study to enrich selenium mainly in terms of its polysaccharides. Such selenized polysaccharides are expected not only to reduce the risk of toxicity for humans caused by the supplementation of inorganic selenium, but also retain the pharmacological activities of polysaccharides [43]. In recent years, polysaccharides from fungi have been intensively excavated for the treatment of numerous diseases, including regulation of human immunity [44], oxidation resistance [45], liver protection, radiation resistance, fatigue resistance, treatment of tumorigenic diseases, etc. [46]. As for polysaccharide from *A. camphorata*, it has been proved to significantly inhibit the differentiation of adipose precursor cells and lipogenesis, and it has therapeutic potential for obesity and related metabolic disorders [47]. A unique polysaccharide component purified from *A. camphorata* elicits its anti-tumor effect by promoting a Th1-dominant state and killer activities [48]. In a Parkinson’s disease mouse model constructed by 6-hydroxydopamine, *A. camphorata* polysaccharide showed good anti-neuroinflammatory effect [49]. Recent data have also suggested that β-D-glucans existing in *A. camphorata* exhibit a strong activity for scavenging free radicals and have been explored as novel potential antioxidants [50]. In terms of antioxidant activity, *G. lucidum* was proved to have a scavenging effect on DPPH radical, which increased with increasing concentration (50–250 mg/mL), showing the highest radical scavenging rate of 72.24% [51]. The scavenging rate for ABTS of *G. lucidum* was 98.68% at the concentration of 5 mg/mL, but the radical scavenging rate was only about 20% at 2 mg/mL [52]. Compared to *A. camphorata* in this study, the antioxidant activity of *A. camphorata* polysaccharides without selenium (51.9%), which was similar to that of *G. lucidum,* was significantly improved by selenium enrichment to 84.2% at a low dosage concentration of 2.0 mg/mL. Considering that selenium-containing catalysts are generally regarded as antioxidants that can protect organisms against reactive oxygen species, our results show that Se-enriched polysaccharides from *A. camphorata* could better meet the sophisticated antioxidation demands, representing a bright prospect for food and pharmaceutical application.

## 4. Materials and Methods

### 4.1. Materials

*A. camphorata* (deposit number: CGMCC 5.906) was a gift from Professor Yang Jianguo of the University of Sydney. Taq polymerase, DNA marker, Fungus Genomic DNA Purification Kit, and primers were purchased from Sangon Biotech (Shanghai) Co., Ltd., China.

### 4.2. Identification of A. camphorata

*A. camphorata* was cultured in a PDA solid medium at 25 °C for 15 d. DNA was extracted using the Fungus Genomic DNA Purification Kit. The extracted DNA was used as the template, and the ITS region was amplified by PCR using the primers of ITS1 (5′-TCCGTAGGTGAACCTGCGG-3′) and ITS4 (5′-TCCTCCGCTTATTGATATGC-3′). The amplified ITS regions were sequenced by Sangon Biotech (Shanghai) Co., Ltd., China. Then, the alignment of the genetic sequences was obtained, and phylogenetic analysis was performed using the MEGA 11 with the neighbor-joining (NJ) method.

### 4.3. Selenium-Enriched Fermentation of A. camphorata

*A. camphorata* was inoculated on the inclined plane of the PDA solid medium at 25 °C for 15 days. The mycelia in the proliferation phase were picked, translocated to a liquid seed culture medium, and shaken at 150 rpm and 25 °C for 5 days. The seed broth was transferred into a fermentation medium containing Na selenite in various concentrations, and it was shaken at 150 rpm and 25 °C for 10 days. The cultivation medium consisted of glucose at 40 g/L, peptone at 10 g/L, K_2_HPO_4_ at 0. 5 g/L, MgSO_4_ at 0.5 g/L, and different concentration Na_2_SeO_3_ (a serial concentration of Se (IV): 0, 5, 10, 20, 40, and 80 mg/L). The working solution of selenium was prepared by dissolving 0.217 g of Na_2_SeO_3_ in 100 mL of deionized water (concentration 1000 mg Se (IV)/L). The aqueous solutions of selenium were prepared so that the final amount of selenium in 100 mL was in the range of 5–80 mg/L. All reagents were sterilized at 121 °C for 20 min.

The cultivation medium volume was two-fifths of shake flask. In order to confirm the conditions of selenium-enriched fermentation, we compared the culture temperature (25 °C and 30 °C), liquid filling volume (50 mL, 75 mL, 100 mL, and 125 mL), and rotational speed (100, 150, and 200 rpm).

Selenium-enriched *A. camphorata* was washed with ultrapure water 3 times to remove residual medium and freeze-dried to weight constancy to determine the dry weight; then, it was crushed and screened through an 80-mesh sieve.

### 4.4. Total Se Determination in A. camphorata

Selenium was determined by LC-AFS (Model No. 6500), manufactured by Beijing Haiguang Instrument Co., Ltd., (Beijing, China) and the Mars6 microwave digestion system manufactured by the American CEM Corporation (Matthews, NC, USA).

The digestion of the sample via mixed acids converted all forms of selenium into the Se (VI) form, and then it was reduced to the tetravalent Se (IV) form by hydrochloric acid to form H_2_Se under the influence of reducing agents such as NaBH_4_ or KBH_4_. Hydrides were carried by a carrier gas (Ar_2_) to an atomizer for atomization. Under the illumination of the selenium hollow cathode lamp, Se was excited from the ground state to a high energy state and then deactivated back to the ground state to emit fluorescence with a characteristic wavelength. The sample was digested according to the PRC Ministry of Health National Standard Method (2017).

The method for the determination of total Se in the mycelia is described below [53]. To a 4 mL mixture of nitric acid, 0.01 g of mycelian powder was added. The mixture was heated on an electric heating plate (165 °C) until white smoke was generated, and the remaining liquid volume was about 0.2 mL. After cooling down to room temperature, 4 mL of concentrated hydrochloric acid was added to the digestion tank, and the solution was heated until it became colorless and white smoke appeared. After the solution was cooled again, the solution was transferred to a 50 mL volumetric flask, bringing it up to the full volume by 5% HCl. The detecting conditions for total selenium by a Hydride Generation-Atomic Fluorescence Spectrometer are shown in Table 5.

**Figure 8:**
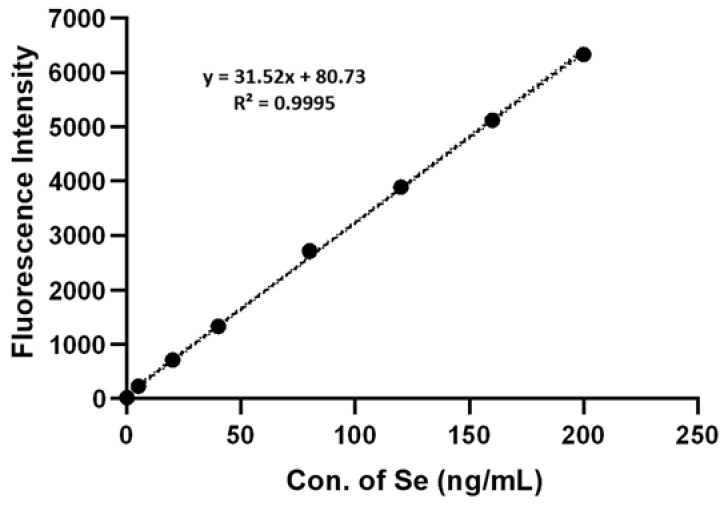
Selenium Standard Curve determined by HG-AFS.

In order to determine total Se in *A. camphorate*, a known quantity of Se was added using a 200 ng/mL sodium selenite solution. The concentrations of Se were 200, 160, 120, 80, 40, 20, and 10 ng/mL, respectively. The samples and blank control were analyzed by HG-AFS with the parameters shown in Table 5. With the fluorescence intensity as the y-coordinate and the concentration of selenium as the x-coordinate, a selenium standard curve was plotted, as shown in Figure 8.

### 4.5. Calculation of Selenium-Enriching Rate

We characterized the Se-enriching capacity of *A. camphorata* mycelia by calculating the utilization rate of inorganic selenium with the following formula:

$$\mathrm{Se}-\mathrm{enriching}\;\mathrm{rate}\;(\%)=\frac{C\times W}{C_{0}}\times 100\%$$

where C is the content of total selenium in *A. camphorata*, W is the dry weight of mycelia, and C_0_ is the content of inorganic selenium added into medium.

### 4.6. Inorganic Selenium Determination in A. camphorata

The methods for the determination of inorganic selenium, as described by Nakaguchi et al. [54,55], followed these steps: dried selenium-enriched *A. camphorata* powder was extracted with 0.6 mol/L hydrochloric acid (1:50, *w*/*v*) at 70 °C for 2 h, and cooled down to room temperature. The extracting solution was collected by centrifugation. The filtrate was collected by centrifugation and transferred together with 3 mL cyclohexane to a separatory funnel to perform liquid–liquid extraction. Then, the aqueous phase was the inorganic selenium solution. The samples and blank control were analyzed HG-AFS using the parameters shown in Table 5.

### 4.7. Calculation of Organic Selenium Content in A. camphorata

There is no national standard on the determination of organic selenium; according to the total selenium content and the inorganic selenium content, the determination of organic selenium was calculated by the subtraction method, using the following formula: “Concentration of organic Se = Concentration of total Se—Concentration of inorganic Se”.

### 4.8. Extraction of Polysaccharide and Determination of Selenium Polysaccharide Content in A. camphorata

Dried selenium-enriched *A. camphorata* powder was defatted with 95% (*v*/*v*) ethanol at reflux for 1.5 h three times. After filtration, the precipitate was extracted with deionized water (1:20, *w*/*v*) at 100 °C for 2 h. Following centrifugation, the supernatant was collected and concentrated. The obtained concentrate was treated with three volumes of ethanol for precipitation at 4 °C for 12 h. The sediment was collected by centrifugation and freeze-dried to weight constancy to prepare the crude polysaccharide. The crude polysaccharide was deproteinized through papain hydrolysis at 70 °C for 2 h; then, the Sevage method was applied for 3 times until there was no precipitation. Subsequently, the polysaccharides were further purified via ionexchange (HiTrap Q FF, 1 mL) and gel filtration chromatography (Superdex 200 Increase 10/300 GL). The phenol–sulfuric acid method, meanwhile, was used for real-time absorbance measurement of the collected samples at 490 nm. Then, the polysaccharide fractions were purified by concentration, dialysis, and freeze-drying. Finally, the concentration of selenium in the polysaccharide samples was analyzed by HG-AFS using the parameters shown in Table 5.

In order to verify the necessity of fermentation for binding selenium with *Antrodia camphorata* polysaccharide, we dissolved selenium-free Antrodia camphorata polysaccharide 2 mg in 10 mL of the sodium selenite solution (10 mg/L, concentration selenium-enriched fermentation), and the mixture was incubated at 150 rpm and 25 °C for 5 days; inorganic selenium was extracted by hydrochloric acid, and the inorganic and total selenium contents were determined by LC-AFS. The samples and blank control were analyzed by HG-AFS using the parameters shown in Table 5, and the negative control was the sodium selenite solution without polysaccharide.

### 4.9. Extraction of Total Soluble Protein and Determination of Selenoprotein Content in A. camphorata

Dried selenium-enriched *A. camphorata* powder was extracted with 0.25 mol/L NaOH (1:10, *w*/*v*) at 50 °C for 4 h. Following centrifugation, the supernatant was collected, the precipitate was extracted by repeating the above steps, and the supernatant was merged. Ammonium sulfate was used for protein precipitation, and (NH_4_)_2_SO_4_ was added until reaching a saturation rate of 75% for precipitation at 4 °C for 12 h. The sediment was collected by centrifugation and resolubilized in a 20 mmol/L Tris-HCl buffer (pH 8.0), and the solution was dialyzed at 4 °C for 48 h (MWCO 3500 Da). The dialysate was freeze-dried to prepare total soluble proteins. The protein sample and blank control were analyzed by HG-AFS using the parameters shown in Table 5.

### 4.10. In Vitro Antioxidant Activity of Polysaccharides

The in vitro antioxidant activity of *A. camphorata* polysaccharides was evaluated by the ABTS and DPPH radical scavenging experiments.

In the ABTS radical removal experiment, potassium persulfate oxidized ABTS to generate blue-green free radical ABTS^+^·, which had a very strong absorption peak at 415 nm. The solution color became lighter due to the reaction of antioxidant with ABTS^+^·, reflecting its ability to remove ABTS radicals [56]. The experiment of eliminating ABTS free radicals was modified according to the literature [57] as follows: 7.4 mmol/L ABTS solution and 2.6 mmol/L persulphuric acid solution with the same volume were mixed evenly, incubated in the dark for 16 h, and then diluted until the absorption value at 734 nm was 0.70 (±0.02) to obtain the ABTS solution for later use. An amount of 2 mg/mL of Vc (L-ascorbic acid) solution and the same amount of polysaccharide solution were accurately prepared and diluted to 1, 0.5, 0.25, 0.125 and 0.0625 mg/mL step by step, respectively. A total of 250 μL of the above solutions was added to 5 mL of ABTS^+^·solution, mixed quickly, and incubated for 30 min at room temperature in the dark; then, their absorbance at 734 nm was measured after the reaction. In this experiment, 250 μL of the sample solution with 5 mL of deionized water was taken as the negative control, and 250 μL of deionized water with 5 mL of the ABTS^+^· solution was taken as the blank control. The experimental treatment methods of the blank control group and the negative control group were the same. Each experiment was repeated 3 times to obtain the average value. The radical scavenging rate was calculated using the following formula.

$$\mathrm{Scavenging}\;\mathrm{activity}\;(\%)=\left(1-\frac{A_{x}-A_{1}}{A_{0}}\right)\times 100$$

where A_0_ is the absorbance of the blank control group; A_1_ is the absorbance of the negative control group; and A_x_ is the absorbance of the sample.

The polysaccharide solution could react with DPPH free radicals to make the purple become lighter, and there was a dose–effect relationship between the degree of color change and the free radical scavenging ability of polysaccharide [56]. The experiment of scavenging DPPH free radicals, as described in the literature [58], was slightly modified as follows: 2 mg/mL of Vc solution and the same amount of polysaccharide solution were precisely prepared and diluted to 1, 0.5, 0.25, 0.125 and 0.0625 mg/mL, respectively. Then, 1mL of each of the above solutions was added to 1 mL of the DPPH solution (0.125 mg/mL), mixed quickly, and incubated in the dark for 30 min at room temperature. After the reaction, the absorbance was measured at 517 nm. In the experiment, 1 mL of the sample solution and 1 L of absolute ethanol were used as the negative control group, and 1mL of deionized water and 1 mL of the DPPH solution (0.125 mg/mL) were used as the blank control group. The experimental treatment methods of the blank control group and negative control group were the same. Each experiment was repeated 3 times to obtain the average value. The radical scavenging rate was calculated according to the above formula.

### 4.11. Statistical Analysis

The data were expressed as the mean ± standard error of measurements from the triplicate experiments. One-way analysis of variance (ANOVA) was used to test for significance of differences, using SPSS 20.0. *p*-values less than 0.05 were considered significant.

## 5. Conclusions

This research determined the optimal conditions of submerged fermentation of *A. camphorata* for selenium enrichment: 10 mL of 5-day-old seed broth was transferred into 100 mL of the fermentation medium (glucose at 40 g/L, peptone at 10 g/L, K_2_HPO_4_ at 0.5 g/L, and MgSO_4_ at 0.5 g/L) in a 250 mL shake flask with an initial concentration of selenite at 10 mg/L, and then cultured at 25 °C for 10 days at 150 rpm. Based on the analysis by HG-AFS, the total selenium content in *A. camphorata* was 1281.3 µg/g, and the organic selenium content was 1128.5 µg/g, accounting for 88.1% of the total selenium content. Selenium polysaccharide was the main pattern of organic selenium existing in selenium-enriched *A. camphorata*, accounting for 61.5% of the organic selenium. Four polysaccharide fractions, ACP I, ACP II, ACP III, and ACP IV, were separated by chromatography and determined by the phenol–sulfuric acid method and LC-AFS. It was shown that the ACP II fraction was the major fraction of Se-polysaccharide; nevertheless, the ACP IV fraction had the highest content of selenium.

Although the time of submerged fermentation of *A. camphorata* in a shake flask was long (~15 days), this culture significantly reduced the time needed for fruiting body cultivation. Combined with its good reproducibility, submerged cultivation would be the optimum technique for *A. camphorata* growth at an industrial level. Based on its strong selenium-enrichment capacity as revealed by our study, the nutritive and pharmaceutical values of *A. camphorata*, a traditional edible and medicinal mushroom, have been further improved. Further study on the bioactivity and structural information of selenium polysaccharide of *A. camphorata* will contribute to the knowledge of *A. camphorata* and broaden its application as a selenium-biofortification method.

## Figures and Tables

**Table 1:** *A. camphorata* mycelial growth at different temperatures in liquid culture.

Temperature (°C)	Dry Weight (g/L)	Conc. of Se (µg/g)
25	1.55 ± 0.06 ^a^	1156.9 ± 49.1 ^a^
30	1.45 ± 0.07 ^a^	872.0 ± 80.6 ^b^

Note: values with superscript letters a and b are significantly different across columns (*p* < 0.05).

**Table 2:** Contents of inorganic selenium and organic selenium.

Conc. of Total Se (µg/g)	Conc. of Inorganic Se (µg/g)	Conc. of Organic Se (µg/g)	Percentage of Organic Se (%)
1281.3 ± 105.1	152.8 ± 20.3	1128.5 ± 87.4	88.1

**Table 3:** Composition of organic selenium.

	Se-Polysaccharide	Selenoprotein
Content of polysaccharide/protein (mg/g dry biomass)	66.3 ± 3.9	58.2 ± 4.3
Content of selenium (µg Se/g Se-polysaccharide or selenoprotein)	694.7 ± 50.3	140.2 ± 9.3
Percentage of organic selenium (%)	61.5	12.4
Percentage of total selenium (%)	54.2	10.9

**Table 4:** Yield and content of polysaccharides and selenium of ACP I, ACP II, ACP III, and ACP IV.

Fraction	Yield (%)	Content of Selenium (µg/g)
ACP I	13.14	45.82 ± 7.92
ACP II	45.57	176.44 ± 20.45
ACP III	6.43	70.87 + 6.98
ACP IV	19.29	202.35 ± 14.71

**Table 5:** Hydride Generation-Atomic Fluorescence Spectrometer Detection Conditions.

Detection Parameters	Operating Conditions
Negative high voltage (V)	250
Lamp current (mA)	80
Carrier gas	Argon
Carrier gas flow (mL/min)	300
Shielding gas flow (mL/min)	800
Reading time (s)	16
Delay time (s)	6
Carry current	0.6 mol/L hydrochloric acid
Reducing agent	Potassium borohydride concentration 2%, contain 0.5% NaOH

## Data Availability

Data are contained within the article.
